# Psychosocial Correlates of Reactive and Proactive Aggression among Protesters during the Social Movement in Hong Kong

**DOI:** 10.3390/ijerph19084679

**Published:** 2022-04-13

**Authors:** Annis Lai Chu Fung

**Affiliations:** Department of Social and Behavioural Sciences, City University of Hong Kong, Hong Kong, China; annis.fung@cityu.edu.hk

**Keywords:** reactive aggression, proactive aggression, moral disengagement, protest, social movement, Hong Kong

## Abstract

This pioneering study examined how psychosocial factors predicted reactive and proactive aggression among adolescents and young adults in Hong Kong during the Anti-Extradition Bill Movement. A total of 1027 local secondary and tertiary students (578 male, 449 female) aged from 12 to 25 years (*M* = 16.95, *SD* = 3.30) completed a questionnaire measuring political participation and attitudes, victimization experiences, aggression, life satisfaction, moral disengagement, and psychopathic traits. ANCOVA and multiple linear regression analyses were performed. The results revealed that compared with non-protesters, protestors had more negative traits and poorer well-being (higher levels of reactive aggression, moral disengagement, narcissism, and impulsivity; lower life satisfaction; more experiences of victimization by strangers related to political disputes). Nonetheless, protesters had similar psychosocial correlates of reactive and proactive aggression when compared to the non-protesters. Among the protesters, reactive aggression was positively predicted by anger towards the government, moral justification, diffusion of responsibility, impulsivity, and narcissism and negatively predicted by satisfaction with the government, advantageous comparison, and dehumanization. Furthermore, proactive aggression was positively predicted by narcissism, euphemistic language, and advantageous comparison and negatively predicted by moral justification. The implications of the findings for psychotherapy, school education, parenting, and social policies are discussed.

## 1. Introduction

The Anti-Extradition Bill Movement (the Movement) in Hong Kong was sparked off by the society’s concerns towards the Fugitive Offenders and Mutual Legal Assistance in Criminal Matters Legislation Bill (the Bill) introduced by the government in 2019 [1]. Though the Bill was intended to enter into extradition agreements with Mainland China and Taiwan, some Hong Kong citizens were worried that they might be apprehended in Mainland China and be subjected to its jurisdiction. From June 2019 onward, millions of Hong Kong people marched on the streets to seek the withdrawal of the Bill [2], but as the government refused to cater for their demands, the Movement continued to expand and led to social and political unrest involving escalated levels of violence and delinquency in Hong Kong, subsequently becoming the focus of global attention. Protesters displayed moderate to extreme levels of aggressive behavior when demonstrating to make their political demands. Subsequently, as COVID-19 hit Hong Kong and the prohibition on gathering was imposed by the government in March 2020 [3], the number of protests started to reduce and was further diminished when the National Security Law came into effect in Hong Kong on 30 June 2020 [4].

From June 2019 to February 2021, over 10,200 people were arrested and more than 2500 people were charged concerning the protests, most of whom ranged in age from teenagers to young people in their early twenties [5]. This trend in arrests and charges brought the issue of youth aggression to the public’s attention.

Aggressive behaviors in young people are related to their psychological development, quality of life, and social and cultural environments. Aggression commonly presents in physical, verbal, relational, and online forms, and it is essential to understand the intent and purposes of these forms of aggression to tackle the issue effectively. The literature defines two subtypes of aggression, reactive and proactive, each of which has distinct underlying motives and functions [6,7].

Studies have consistently reported that reactive aggression is correlated with internalizing problems, anger, hostility, impulsivity, and poor social skills, whereas proactive aggression is correlated with narcissism, delinquency, disruptive behaviors, and the initiation of fights [8,9,10]. However, no study has examined the differential psychosocial correlates of reactive and proactive aggression among adolescents and young adults in the context of political and social turmoil. Our study fills this gap in the literature by investigating how different psychosocial factors distinctively predicted reactive and proactive aggression among local secondary and tertiary students in the 2019–2020 Hong Kong protests. The results provide practical insights into intervention strategies for reducing their aggressive behaviors and helping these individuals restore their quality of life amid and after the social unrest.

### 1.1. Distinguishing between Reactive and Proactive Aggression

Many studies have categorized aggressive behaviors into two subtypes based on their function and motivation: (i) reactive aggression, which refers to a vengeful and defensive response to a perceived threat, driven by a short temper; and (ii) proactive aggression, which is regarded as a cold-blooded and planned behavior exercised with the intent to gain anticipated benefits [11].

In the social information-processing model, both reactive and proactive aggression are associated with cognitive distortions at different stages. Reactive aggression is associated with deficient encoding and interpretation of social cues, whereas proactive aggression is linked to the inaccurate evaluation of the potential consequences of such behavior at the response-decision stage [12]. Due to a hostile attribution bias, reactive aggressors are more likely to select negative cues from the social environment and misjudge others’ ambiguous acts as hostile [13]. A failure to accurately encode and interpret others’ intentions through facial and verbal cues may increase the risk of executing an angry response [14].

In contrast, proactive aggressors tend to overestimate their power and ability to perform aggressive acts and underestimate the negative consequences of their behavior. A positive evaluation of the response outcomes and a sense of superiority over others may encourage proactive aggressors to use aggression instrumentally, allowing them to take advantage of others and obtain rewards such as money and dominance [15]. Hence, reactive aggressors are often regarded as “hot-tempered” and proactive aggressors as “cold-blooded” [16]. The distinct functions and features of reactive and proactive aggression suggest that different levels of psychological and social competence shape the two subtypes of aggressive behavior.

### 1.2. Psychosocial Correlates of Reactive and Proactive Aggression

Previous meta-analytical findings suggest that adolescents who display reactive aggression are predisposed to a lower quality of life concerning internalizing problems (e.g., anxiety and depressive symptoms), emotional dysregulation, impulsivity, low peer acceptance, and peer victimization than are those who display proactive aggression [17]. Several studies have shown that reactive aggression is associated with heightened anger expression, anxiety, impulsivity, hyperactivity, schizotypal traits (e.g., paranoid ideation and social anxiety), and poor interpersonal skills [8,10,18]. In contrast, proactive aggression is often associated with narcissism, externalizing problems, such as delinquency and antisocial behavior, and poor prosocial behavior [9,10,19]. The potential psychosocial factors contributing to reactive and proactive aggression in the context of the 2019–2020 Hong Kong protests, at the individual level, are discussed in the following sections.

#### 1.2.1. Anger

Reactive aggression can be an angry reaction triggered by a perceived threat or provocation [13]. Adolescents with reactive aggression, characterized by a hostile attribution bias, selective attention to negative cues, and poor emotional regulation, are more likely to display high levels of emotional reactivity and irritability [20]. Moreover, a study on the short-term consistency of aggression and its correlates in second-grade children found that reactive aggression was positively associated with dysregulated anger expression; this association remained stable over the one-year study period, whereas proactive aggression remained unrelated to anger expression throughout the study period [8].

The protesters and their supporters displayed much anger towards the police and the Hong Kong government. The protesters considered themselves victims of the excessive use of force by the police [21], with slogans, such as “never forget, never forgive”, “Hongkongers, revenge”, and “death to the families of the evil police” frequently seen in demonstrations, graffiti, and propaganda [22]. The retaliation against the police and parties with contrary political views reflected reactive aggression.

#### 1.2.2. Dissatisfaction with the Government

Shek [21] argued that a long-standing dissatisfaction with the Hong Kong government contributed to the outburst of the protests in 2019 and 2020. Some people in Hong Kong, especially young people, were not satisfied with the political system of Hong Kong and thought the government had not been working effectively after the handover. One of the “five demands” of the protesters was dual universal suffrage [23]. Dissatisfaction with the government may contribute to proactive aggression in protesters to achieve their political demands but may also trigger anger and frustration, leading to reactive aggression [24].

#### 1.2.3. Moral Disengagement

A meta-analysis of 27 studies revealed that moral disengagement was positively associated with aggressive behaviors in children and adolescents aged 8–18 years, with a mean effect size of 0.28 [25]. The social cognitive theory of moral agency, proposed by Bandura and colleagues [26], posits that although moral reasoning and standards provide the grounds for moral conduct, individual behavior is determined by self-regulatory functions that influence the individual’s engagement or disengagement in moral conduct. According to this theory, aggressive behavior can be further promoted by an individual’s tendency to morally disengage, justify, or rationalize his/her behavior through several cognitive mechanisms: (i) moral justification (i.e., justifying a negative behavior as serving a moral or social function), (ii) euphemistic labeling (i.e., using sanitizing language to rationalize a negative behavior), (iii) advantageous comparison (i.e., comparing a negative behavior to worse acts to make it sound less harmful), (iv) diffusion of responsibility (i.e., distributing responsibility by emphasizing group decision-making and collective action), (v) displacement of responsibility (i.e., viewing a legitimate authority as responsible for a negative behavior instead of oneself), (vi) distorting consequences (i.e., disregarding negative consequences and emphasizing the positive outcomes of a harmful act), (vii) attribution of blame (i.e., viewing the victim as deserving of or responsible for the harmful act), and (viii) dehumanization (i.e., depriving the victim of positive human qualities to disregard moral concerns) [27].

A predisposition to moral disengagement, mediated by a lack of empathic concern and perspective-taking, is associated with higher levels of peer aggression [28]. These mechanisms allow individuals to exhibit self-serving behavior that violates their moral standards without triggering self-evaluative emotional reactions such as remorse and guilt [29]. Therefore, it is found that displays of proactive aggression featuring a callous lack of emotion and empathy toward others’ suffering are specifically linked to or mediated by moral disengagement mechanisms. It has been suggested that these mechanisms become more stable over time as an individual increasingly commits transgressions in the pursuit of self-interest [29]. This also indicates that moral disengagement and proactive aggression may be mutually reinforcing as people engage in more aggressive behavior with positive outcomes.

During the 2019–2020 Hong Kong protests, beliefs, such as “disobeying the law to obtain justice is acceptable” and “violence is sometimes necessary under certain circumstances” were held by some protesters and the public [21]. These are examples of moral justification, but the kinds of mechanisms used to rationalize their aggressive and illegal acts are not clear.

#### 1.2.4. Life Satisfaction

Shek [21] argued that a drop in life satisfaction among young people in Hong Kong was one of the predisposing factors of the 2019–2020 Hong Kong protests. Aggressive behavior has been associated with maladjustment spanning adolescence and adulthood, such as internalizing problems (e.g., anxiety and depressive symptoms), in reactive aggressors [30]. A study conducted on a sample of 1510 adolescents aged 12–17 years found that personal maladjustment and aggressive behaviors were associated with low levels of self-esteem, life satisfaction, and empathy and with high levels of depressive symptoms, stress, and loneliness [31]. A recent longitudinal study found that life satisfaction had decreased, and hopelessness had increased in Hong Kong adolescents from 2009 to 2015 [32]. During social unrest, dissatisfied young people are more likely to exhibit reactive aggression because of their frustration with life, or to exhibit proactive aggression to gain a sense of satisfaction.

#### 1.2.5. Victimization Experiences

The Movement polarized Hong Kong people with different political views, causing conflict within families, schools, and social groups [21]. Some people attacked others verbally, socially, physically, or online. More school bullying incidents occurred due to differences in political views [33]. The Education Bureau received 346 bullying case reports from primary and secondary schools during the 2019–2020 school year, which was a 10-year high and a 53% increase over the 226 cases reported in 2018–2019 [34].

A meta-analysis revealed that externalizing problems, including aggression, are both antecedents and consequences of peer victimization in youth; in other words, peer victimization predicts aggressive behaviors and those who behave more aggressively are more likely to be attacked by their peers [35]. Reactive aggressors are prone to dramatic emotional reactions and disruptive behaviors in school, which can lead to peer rejection [14,36]. Aggravated victimization, in turn, increases their reactive aggression as they try to defend themselves from victimization, leading to a vicious cycle [35,37].

Therefore, we expect that during the political and social turmoil, repeated victimization related to political disputes by peers increased adolescents’ tendency to use reactive aggression to defend themselves or retaliate against perceived threats or provocation. Moreover, during the protests, there were incidents of conflict, assaults, fights, or vigilantism among people of opposing political stances [38]. We expect that victimization by strangers also increased the tendency to use reactive aggression in the young protesters.

### 1.3. Psychopathic Traits

Aside from short-term cognitive and emotional conditions, certain traits may predispose adolescents to use aggression. Psychopathic traits in adolescence are associated with childhood conduct problems, juvenile delinquency, and adult criminal behaviors [39].

#### 1.3.1. Narcissism

Previous studies have revealed that children with high narcissism display consistently aggressive behavior throughout their development [40]. Likewise, aggressors have been shown to have higher levels of narcissism than non-aggressors [41]. A recent study revealed that young adults with high levels of narcissism were more likely to display proactive aggression, whereas those with lower self-esteem were predisposed to hostility and anger, and thereby displayed more reactive aggression [42]. However, another study found that narcissism was associated with both subtypes of aggression and predicted severe conduct problems [43]. Therefore, although it is generally accepted that narcissism is a positive predictor of proactive aggression, its association with reactive aggression remains unclear.

#### 1.3.2. Impulsivity

Several studies have found that impulsivity is uniquely associated with reactive but not proactive aggression in children and adolescents [43,44]. These findings suggest that reactive aggressors are less capable of emotional regulation and behavioral inhibition than proactive aggressors. Nevertheless, Orue and Andershed [45] provided evidence that the interplay between impulsivity and narcissism may predict the development of proactive aggression. Although impulsivity was not directly associated with proactive aggression, adolescents who showed high levels of narcissism and impulsivity displayed more proactive aggression than those who had neither of these traits. Overall, we expect impulsivity to be more strongly associated with reactive aggression, which is often an immediate emotional reaction, than with proactive aggression, which serves to attain personal goals and rewards.

### 1.4. Present Study

Although specific psychosocial correlates of reactive and proactive aggression are identified in the literature, no studies have examined how these factors correlate with the two subtypes of aggression in the context of political and social turmoil. Our study explores how different psychosocial factors distinctively predicted reactive and proactive aggression among local secondary and tertiary students in the 2019–2020 Hong Kong protests. The findings provide insights into the quality of life and the underlying psychosocial mechanisms of aggressive and illegal acts among adolescents and young adults in the Movement. Hence, intervention could be adjusted to suit the particular needs of the protesters.

### 1.5. Hypotheses

(I) Compared with non-protesters, the protesters in the 2019–2020 Hong Kong protests have higher levels of reactive aggression, proactive aggression, anger towards the Hong Kong government, moral disengagement, victimization experiences, narcissism, and impulsivity, and lower levels of satisfaction with the Hong Kong government and life satisfaction.

Among the protesters, (II) anger towards the Hong Kong government positively predicts the level of reactive aggression but not of proactive aggression; (III) satisfaction with the Hong Kong government negatively predicts the levels of reactive aggression and proactive aggression; (IV) moral disengagement mechanisms positively predict the level of proactive aggression but not of reactive aggression; (V) life satisfaction negatively predicts the levels of reactive aggression and proactive aggression; (VI) victimization experiences related to political disputes, either by peers or by strangers, positively predict the level of reactive aggression but not of proactive aggression; (VII) narcissism positively predicts the level of proactive aggression but not of reactive aggression; and (VIII) impulsivity positively predicts the level of reactive aggression but not of proactive aggression.

## 2. Materials and Methods

### 2.1. Participants

One thousand and twenty-seven participants (578 male, 449 female) aged 12–25 years (*M* = 16.95, *SD* = 3.30) were recruited. The sample consisted of 622 secondary students (419 male, 203 female) aged 12–20 years (*M* = 14.66, *SD* = 1.65) from three local secondary schools and 405 tertiary students (159 male, 246 female) aged 17–25 years (*M* = 20.48, *SD* = 1.66) from 29 local tertiary institutions. The selection criteria were being (i) a Hong Kong permanent resident, (ii) aged 12–25 years, and (iii) a student at a local secondary school or tertiary institution.

### 2.2. Procedures

Ethical approval was granted by the Research Committee at the City University of Hong Kong. The data collection period lasted from June 2020 to November 2020. All participants were informed that the data collected would be kept anonymous and confidential and strictly protected from disclosure to any unauthorized parties.

#### 2.2.1. Secondary Students

The author recruited five secondary schools in Hong Kong, one from each of the five geographical constituencies (Kowloon East, Kowloon West, New Territories East, New Territories West, and Hong Kong Island) to participate in a survey about their students’ views, emotions, and behaviors regarding the Anti-Extradition Bill Movement in Hong Kong. The schools were randomly selected from a list of 50 participating schools in the author’s previous research projects on child and adolescent aggression [46]. Eight schools were invited before reaching the target of five. Two schools withdrew from the study before the data collection. Finally, three schools (one from New Territories East, one from New Territories West, and one from Kowloon West) agreed to participate, and permission was obtained from the schools’ administrations. All secondary students from grades one to five were then invited to complete a questionnaire (about 1300 students in total). Parental consent and students’ assent were obtained before the participating students completed the questionnaire. The students completed the questionnaire, either in an electronic or paper-and-pencil format, in groups of about 20 in a classroom setting and under the supervision of a research assistant. The students were not allowed to discuss the contents of the questionnaire while filling it out.

#### 2.2.2. Tertiary Students

The author distributed an open invitation to all students attending local tertiary institutions through online promotions on different social media platforms, including Instagram, Facebook, Hong Kong Golden Forum, and the LIHKG forum. A cash incentive of HKD 300 was provided to each participant. The students individually completed an electronic questionnaire using either a tablet or laptop provided by the author, in a room at the City University of Hong Kong. Before participation, the participants provided proof of their student identity (e.g., student card), but no personally identifiable information was recorded.

### 2.3. Measures

The survey consisted of demographic items, questions about political participation and attitudes, victimization experiences, and psychological measures of aggression, moral disengagement, life satisfaction, and psychopathic traits.

#### 2.3.1. Political Participation and Attitudes

The respondents were asked whether they had participated in the Anti-Extradition Bill Movement in any form, ranging from moderate (e.g., peaceful demonstration, non-cooperation movement, Lennon Wall, human chain, Yellow economic circle) to radical (e.g., vandalism, violence, petrol bombing, arson) acts. Regarding their political attitudes, the respondents indicated how angry they were with the actions taken by the government in response to the Anti-Extradition Bill Movement on an 11-point scale (0 = not at all, 10 = extremely angry) and also indicated how satisfied they were with the overall performance of the Hong Kong SAR government on an 11-point scale (−5 = very dissatisfied, 5 = very satisfied).

#### 2.3.2. Victimization Experiences

Given the multidimensionality of peer victimization, we instructed respondents to report their victimization experiences on five dimensions, namely physical victimization, verbal victimization, social manipulation, attack on property, and electronic victimization [47,48]. The items were formulated with reference to the Multidimensional Peer Victimization Scale (MPVS; [48]) and the Multidimensional Peer Victimization Scale-Revised (MPVS-R; [47]). Participants indicated how often they had experienced each form of victimization, by peers and by strangers, because of political disputes over the past year (since June 2019 when the Movement started) on an 11-point scale (0 = never, 10 = always). Each item was accompanied by two brief examples (physical victimization: punch, kick; verbal victimization: swear, call me names; social manipulation: leave me out, turn other people against me; an attack on property: damage my property, take my property without permission; electronic victimization: send me a nasty text, doxing). Social manipulation by strangers was not included, as it was self-contradictory. The five items on victimization by peers and the four items on victimization by strangers were averaged to yield scores for victimization by peers (Cronbach’s alpha = 0.91) and victimization by strangers (Cronbach’s alpha = 0.88), respectively.

We also instructed the respondents to complete the following psychological measures based on their condition over the past year (from the start of the Movement in June 2019).

#### 2.3.3. Reactive–Proactive Aggression Questionnaire

The Reactive–Proactive Aggression Questionnaire (RPQ; [9]) is a 23-item self-report questionnaire that measures reactive and proactive aggression. The items were rated on a 3-point scale (0 = never, 1 = sometimes, 2 = often), with 11 items for reactive aggression (e.g., “got angry when others threatened you”) and 12 items for proactive aggression (e.g., “had fights with others to show who was on top”). The Chinese version of the RPQ, previously validated for adolescents in Hong Kong, was used in this study [49]. Cronbach’s alpha was 0.85 for reactive aggression and 0.89 for proactive aggression.

#### 2.3.4. Antisocial Process Screening Device

The Antisocial Process Screening Device (APSD) is a parent-report questionnaire initially used to detect antisocial processes and psychopathy in youth [50]. In the current study, the participants were instructed to evaluate themselves on a 3-point scale (0 = not at all true, 1 = sometimes true, 2 = definitely true), with five items for impulsivity (e.g., “act without thinking of the consequences”) and seven items for narcissism (e.g., “get angry when corrected or punished”). The self-report adaptation was reported in the literature [51,52,53]. The Chinese version of the APSD was used in this study, but the subscale of callous-unemotional traits was not included because of its low reliability [49]. Cronbach’s alpha was 0.64 for impulsivity and 0.75 for narcissism.

#### 2.3.5. Mechanisms of Moral Disengagement Scale

The Mechanisms of Moral Disengagement Scale (MMDS; [26]) is a 32-item questionnaire that assesses moral disengagement in children and adolescents. For each item, the respondents rated their degree of acceptance of moral exoneration on a 3-point Likert scale. The eight disengagement mechanisms (four items each) assessed in this study were moral justification (e.g., “it is alright to fight to protect your friends”), euphemistic language (e.g., “slapping and shoving someone is just a way of joking”), advantageous comparison (e.g., “damaging some property is no big deal when you consider that others are beating people up”), diffusion of responsibility (e.g., “a kid in a gang should not be blamed for the trouble the gang causes”), displacement of responsibility (e.g., “if kids are living under bad conditions they cannot be blamed for behaving aggressively”), distorting consequences (e.g., “it is okay to tell small lies because they don’t really do any harm”), attribution of blame (e.g., “if kids fight and misbehave in school it is their teacher’s fault”), and dehumanization (e.g., “some people deserve to be treated like animals”). For this study, the author translated the MMDS into Chinese through back-translation. Cronbach’s alpha was 0.71 for moral justification, 0.71 for euphemistic language, 0.71 for advantageous comparison, 0.64 for the diffusion of responsibility, 0.74 for the displacement of responsibility, 0.66 for distorting consequences, 0.59 for attribution of blame, and 0.70 for dehumanization.

#### 2.3.6. Multidimensional Students’ Life Satisfaction Scale

The Multidimensional Students’ Life Satisfaction Scale (MSLSS; [54]) is a 40-item scale that measures life satisfaction among students across five dimensions: family (seven items), friends (nine items), school (eight items), living environment (nine items), and self (seven items). All items were rated on a 6-point scale ranging from 1 (strongly disagree) to 6 (strongly agree) to indicate the extent to which the statements accurately described the students’ feelings about their lives [55]. For this study, the author translated the MSLSS into Chinese through back-translation. Cronbach’s alpha was 0.92 for the overall scale (i.e., across all 40 items).

### 2.4. Design

The study was cross-sectional. First, the mean differences between protesters and non-protesters in reactive aggression, proactive aggression, anger towards the Hong Kong government, satisfaction with the Hong Kong government, victimization by peers, victimization by strangers, moral disengagement, life satisfaction, narcissism, and impulsivity were examined (Hypothesis I). The sizes of effect were evaluated using Cohen’s *d_s_*.

Multiple linear regression analyses were then used to test hypotheses II to VIII. Previous studies have reported medium to high correlations, ranging from 0.41 to 0.76, between reactive and proactive aggression [9,11,56,57,58]. Therefore, reactive and proactive aggression were input as covariates in the regression models when predicting the levels of proactive and reactive aggression, respectively, so that the predictors of pure reactive aggression (independent of proactive aggression) and pure proactive aggression (independent of reactive aggression) could be evaluated.

Reactive and proactive aggression regressed along with age, sex (with female coded as the baseline), and proactive/reactive aggression as covariates. Anger towards the Hong Kong government, satisfaction with the Hong Kong government, victimization by peers, victimization by strangers, moral disengagement (moral justification, euphemistic language, advantageous comparison, diffusion of responsibility, displacement of responsibility, distorting consequences, attribution of blame, and dehumanization), life satisfaction, narcissism, and impulsivity were added as predictors, predicting reactive aggression and proactive aggression (two separate models). Multicollinearity was diagnosed using variance inflation factor (VIF) values.

To investigate the differences in the model structure between protesters and non-protesters, multiple group analysis was used. Chi-square difference tests were used to test the differences in model fit between the null models (in which the regression coefficients were assumed to be equal for protesters and non-protesters) and the unconstrained models (in which all regression coefficients were allowed to differ between groups). Alternative models were further tested when the constraints on the significant coefficients were subsequently released one by one, based on the modification indices. The final regression models were evaluated using R^2^ and β.

## 3. Results

### 3.1. Demographics and Descriptive Statistics

Among the participants, 876 (85%) were born in Hong Kong. For the 151 (15%) who were not born in Hong Kong, the average number of years of residence in Hong Kong was 13.66 years (*SD* = 3.71). Most of the participants lived in Kowloon (*n* = 505) and the New Territories (*n* = 468), and 53 lived on Hong Kong Island. Only 417 participants reported their family income (593 reported that they either did not know or were unwilling to disclose), of whom 13 reported a value of less than HKD 10,000 (3%), 88 reported HKD 10,000–19,999 (20%), 96 reported HKD 20,000–29,999 (22%), 69 reported HKD 30,000–39,999 (16%), 48 reported HKD 40,000–49,999 (11%), 103 reported HKD 50,000 or more (24%), and 17 reported receiving comprehensive social security assistance (4%).

#### 3.1.1. Political Participation and Attitudes

Regarding participation in the Anti-Extradition Bill Movement, 276 secondary students (44%) reported that they had participated, 256 (41%) reported that they had not participated, and 90 (15%) did not disclose whether they had participated. Among the tertiary students, 323 (80%) reported that they had participated, 31 (8%) reported that they had not participated, and 51 (13%) did not disclose whether they had participated. Table 1 presents a summary of the demographic characteristics of the participants, divided into protesters, non-protesters, and non-responses.

Analysis of variance (ANOVA) and chi-square tests were used to examine whether participation in the protests was associated with demographic factors, such as age, sex, and place of birth. However, family income was not examined because 58% of the participants did not report it (43% reported they did not know or were not willing to tell; 15% were missing). Since a considerable number of participants (*n* = 141; 14%) did not disclose whether they had participated in the protests, non-responses would be included as one of the categories of protest participation. Results revealed that there were significant differences in age among protesters, non-protesters, and non-responses, *F*(2, 1024) = 75.02, *p* < 0.001. Bonferroni post hoc tests indicated protesters were significantly the oldest, while non-protesters were significantly the youngest. Protest participation was significantly associated with sex, χ^2^(2, *N* = 1027) = 15.88, *p* < 0.001, but not with place of birth, χ^2^(2, *N* = 1027) = 2.79, *p* = 0.25.

Regarding satisfaction with the Hong Kong government, the participants were generally not satisfied with the overall performance of the government, *M* = −3.13, *SD* = 2.40, *t*(1020) = −41.72, *p* < 0.001. Consistently, the participants were generally angry with the actions taken by the Hong Kong government in response to the Anti-Extradition Bill Movement, *M* = 6.64, *SD* = 3.55, *t*(1025) = 59.88, *p* < 0.001.

#### 3.1.2. Victimization Experiences

Regarding victimization related to political disputes, 24% of the participants reported that they had experienced victimization by their peers, 27% reported that they had experienced victimization by strangers, and 37% reported that they had experienced victimization regardless of who the perpetrators were (i.e., indicated 1 or above for any form of victimization). The descriptive statistics for each form of victimization are presented in Table 2.

### 3.2. Correlations

The correlations between the two subtypes of aggression and other psychosocial measures are presented in Table 3. As some of the variables were not normally distributed, Spearman’s correlation was used. In keeping with the previous reports, we observed a medium correlation between reactive and proactive aggression, *r_s_* = 0.44, *p* < 0.001.

### 3.3. Group Differences

As protest participation was significantly associated with age and sex, their effects were controlled in the analyses. Results from 2 × 3 analysis of covariance (ANCOVA) indicated that after controlling for age and sex, there were significant differences in reactive aggression, anger towards the government, satisfaction with the government, victimization (both by peers and strangers), life satisfaction, moral disengagement mechanisms (all eight mechanisms), narcissism, and impulsivity among protesters, non-protesters, and non-responses. The protester group differences were insignificant for proactive aggression, *p* = 0.10.

Results from the Bonferroni post hoc tests indicated that compared with participants who had not participated in the protests, protesters had significantly higher levels of reactive aggression, anger towards the government, moral disengagement mechanisms (all eight mechanisms), victimization by strangers, narcissism, and impulsivity, but significantly lower levels of satisfaction with the government and life satisfaction. The effect sizes were very large for anger towards the government (*d* = 1.68) and satisfaction with the government (*d* = −1.36) but small for the rest (ranging from 0.20 to 0.36).

Compared with non-protesters, the non-responses had significantly higher levels of anger towards the government, moral disengagement mechanisms (all except moral justification, distorting consequences, and attribution of blame), and victimization (both by peers and strangers), but significantly lower levels of satisfaction with the government and life satisfaction. Furthermore, compared with protesters, the non-responses had significantly higher levels of dehumanization and satisfaction with the government, but significantly lower levels of reactive aggression, anger towards the government, narcissism, and impulsivity.

Regarding the sex differences, after controlling for age and protest participation, males had significantly higher levels of proactive aggression, moral disengagement mechanisms (all except diffusion of responsibility), victimization (both by peers and strangers), impulsivity, and narcissism, and significantly lower levels of life satisfaction. However, the sex × protest participation interaction effect was only significant for life satisfaction, *F*(2, 1020) = 3.44, *p* = 0.03, while the rest interaction effects were all insignificant, *p* > 0.05. Bonferroni post hoc tests revealed that females had significantly higher levels of life satisfaction than males among non-protesters and non-responses, while the sex difference was not significant among protesters. Details of the ANCOVAs, post hoc comparisons among the three levels of protest participation, and the effect sizes for the mean differences between protesters and non-protesters are presented in Table 4 and Table 5.

### 3.4. Regression Analyses

As some predictors and criteria were not normally distributed, we used a robust estimator in Mplus, version 7.4 (Muthén & Muthén, Los Angeles, CA, USA), with maximum likelihood parameter estimates with standard errors and a mean-adjusted chi-square test statistic that is robust to non-normality (MLM). In the multiple group analysis, to examine the moderation effects of protest participation (two levels; protesters vs. non-protesters), the model fit of an unconstrained model was first compared with that of a null model. Constraints on the significant paths were then released one by one. Finally, the differences in intercepts between the groups were tested. The Satorra–Bentler scaled chi-square difference test was used because of the MLM estimator. Details of the model comparison are presented in Table 6.

#### 3.4.1. Reactive Aggression

For both protesters and non-protesters, the unconstrained regression model significantly predicted reactive aggression, *R*^2^ = 0.42, *p* < 0.001, and *R*^2^ = 0.43, *p* < 0.001, respectively. Table 7 presents the details of the unconstrained regression model. All VIF values were less than 10, thus showing no signs of multicollinearity.

The Satorra–Bentler scaled chi-square difference test indicated the unconstrained model significantly differed from the null model, *T*(18) = 50.34, *p* < 0.001. According to the modification indices, the constraint on satisfaction with the government was released, *T*(1) = 15.32, *p* < 0.001, followed by impulsivity, *T*(1) = 4.14, *p* = 0.04. However, there were no significant differences in model fit between the intercept-constrained model and the intercept-unconstrained model, *T*(1) = 2.29, *p* = 0.13, suggesting there was no difference in the intercepts of reactive aggression between the protesters and non-protesters.

In the final multiple group model, after controlling for age, sex, and proactive aggression, among both protesters and non-protesters, anger towards the government, moral justification, diffusion of responsibility, impulsivity, and narcissism positively predicted reactive aggression, and advantageous comparison, and dehumanization negatively predicted reactive aggression. Protest participation significantly moderated the path between satisfaction with the government and reactive aggression, in which satisfaction with the government negatively predicted reactive aggression only in protesters, but not in non-protesters. Furthermore, the relation between impulsivity and reactive aggression was stronger in non-protesters than in protesters. The final model explained a 41% variance of reactive aggression in protesters and 43% of that in non-protesters. For the significant predictors, βs ranged from 0.08 to 0.26, indicating a very small to small effect. Table 8 presents the details of the final model.

#### 3.4.2. Proactive Aggression

For both protesters and non-protesters, the unconstrained regression model significantly predicted proactive aggression, *R*^2^ = 0.32, *p* < 0.001, and *R*^2^ = 0.45, *p* < 0.001, respectively. Table 9 shows the details of the unconstrained model. All VIF values were less than 10, giving no indication of multicollinearity.

The Satorra–Bentler scaled chi-square difference test indicated the unconstrained model significantly differed from the null model, *T*(18) = 138.62, *p* < 0.001. According to the modification indices, the constraint on advantages comparison was released, *T*(1) = 28.59, *p* < 0.001, followed by victimization by peers, *T*(1) = 17.50, *p* < 0.001, sex, *T*(1) = 16.90, *p* < 0.001, and lastly, narcissism, *T*(1) = 4.58, *p* = 0.03. Nevertheless, there were no significant differences in model fit between the intercept-constrained model and the intercept-unconstrained model, *T*(1) = 2.83, *p* = 0.09, suggesting there was no difference in the intercepts of proactive aggression between the groups.

In the final multigroup model, after controlling for age, sex, and reactive aggression, among the protesters, euphemistic language, advantageous comparison, and narcissism positively predicted proactive aggression, and moral justification negatively predicted proactive aggression, whereas, among the non-protesters, victimization by peers and advantageous comparison positively predicted proactive aggression, and moral justification negatively predicted proactive aggression. Protest participation had significant moderation effects on the relations between proactive aggression and sex, victimization by peers, advantageous comparison, and narcissism. The final model explained a 31% variance of proactive aggression in protesters and 44% of that in non-protesters. For the significant predictors, βs ranged from 0.09 to 0.27, indicating a very small to small effect. Details of the final model are presented in Table 10.

## 4. Discussion

Our findings indicate that among secondary and tertiary students, those who did participate in the 2019–2020 Hong Kong protests had a lower quality of life than those who did not participate. For example, the protesters had more reactively aggressive behaviors, higher levels of narcissism and impulsivity, lower life satisfaction, and experienced more victimization by strangers related to political disputes. Moreover, the protesters had a larger extent of moral disengagement.

Nonetheless, protesters and non-protesters had similar psychosocial correlates of reactive and proactive aggression. Among the protesters, the level of reactive aggression was positively predicted by anger towards the government, moral justification, diffusion of responsibility, impulsivity, and narcissism, and negatively predicted by advantageous comparison, dehumanization, and satisfaction with the government. Their level of proactive aggression was positively predicted by narcissism, euphemistic language, and advantageous comparison, and negatively predicted by moral justification.

Overall, our findings are broadly consistent with previous findings, suggesting that reactive and proactive aggression are differentially predicted by specific psychosocial factors and follow distinct etiological pathways.

### 4.1. Anger and Dissatisfaction

Protesters’ level of reactive aggression, but not proactive aggression, was predicted by a higher level of anger towards the Hong Kong government, consistent with the notion that reactive aggression can be an angry reaction triggered by a perceived threat or provocation [13]. The protesters reported intense anger towards the police and the Hong Kong government. For example, the death of a university student who fell from a height during a protest in November 2019 inflamed the anger of protesters [59]. More than half of the respondents to a Centre for Communication and Public Opinion Survey [60] did not trust and were not satisfied with the Hong Kong police. Therefore, the protesters and their supporters considered the police force as a threat, and the aggressive behaviors during the protests were thus reactive.

Although non-protesters did not engage in the protests, their level of reactive aggression was also positively predicted by the anger towards the Hong Kong government. A general state of anger, regardless of the causes, would increase the likelihood of reactive aggression [61]. They might exhibit reactive aggression more often in daily life if they were angry at the government.

Satisfaction with the government negatively predicted reactive aggression only in protesters, but not in non-protesters, and its relation with proactive aggression was insignificant. These findings suggest that although some protesters were highly dissatisfied with the Hong Kong government, their aggressive behaviors were mainly the result of frustration and emotional reactivity and were less likely to have been deliberate actions to achieve political goals. Consistently, we found that there was no difference in proactive aggression between protesters and non-protesters.

### 4.2. Moral Disengagement

Reactive aggression was significantly predicted by higher moral justification and diffusion of responsibility but lower advantageous comparison and dehumanization. In contrast, lower moral justification and higher advantageous comparison predicted proactive aggression. These findings suggest that protesters with reactive aggression are more likely to justify their aggressive behaviors as serving moral or social functions and participate in collective violence, whereas those with proactive aggression tend to compare their aggressive acts to even more violent or harmful acts to rationalize their behaviors.

Previous studies have found that adolescents who exhibit more proactive aggression show lower moral concerns when evaluating the consequences of their aggressive behaviors, highlighting their tendency to underestimate the unfairness or harm incurred to their victims [16]. The results imply that such a tendency in proactive aggressors may involve active efforts to disregard the negative costs of their actions by comparing their aggressive behavior to more harmful acts [29]. By discrediting negative consequences, proactive aggressors may bias themselves toward positive outcomes and behave aggressively toward others without guilt.

In contrast, it has been proposed that reactive aggressors can recognize common moral values but lack the social reasoning skills with which to judge whether others have violated such moral standards [62]. In the findings of the present study, reactive aggression was predicted by the moral justification mechanism that allows individuals to justify their aggressive behavior as a personally or socially acceptable means to defend their social reputation or power [29]. This suggests that reactive aggressors could recognize common moral standards but may misuse them to justify their behaviors as reasonable defensive responses, further demonstrating impaired moral reasoning. Poor moral reasoning skills reduce people’s ability to discern the “wrongness” of deviant behavior, such as violence against people and property that can infringe on others’ rights and damage their welfare [63]. In situations that make them feel irritated or threatened, reactive aggressors can be easily triggered and behave aggressively toward others to defend themselves.

Fung [30] argued that adolescents with reactive aggression were more likely to lack a sense of security and to be recruited by triads, manipulated by ringleaders, and become the scapegoats of crimes. Our finding of a positive association between reactive aggression and diffusion of responsibility among the protesters is consistent with this argument. In collective violence, the sense of security is increased and the sense of responsibility is minimized. The use of black bloc tactics in the protests to conceal individual identities further diffused responsibility from individuals to the group.

Nevertheless, our findings indicated that the positive relation between advantageous comparison and proactive aggression was significantly stronger in non-protesters than in protesters. A possible reason is that the violent and illegal behaviors of some protesters became the convenient target of advantageous comparison for non-protesters. Proactively aggressive behaviors were thus thought to be less harmful, compared to the radical acts (e.g., petrol bombing, arson) frequently portrayed in the mass media.

### 4.3. Victimization Experiences

Contrary to our hypothesis, our results indicated that the protesters’ victimization experiences related to political disputes, by peers or by strangers, did not predict the level of either reactive or proactive aggression. A possible reason for this finding is that victimization experiences of various forms, especially related to political disputes, were common among people in Hong Kong during the social unrest in 2019 and 2020. There were conflicts between people who supported and opposed the protest in families, schools, workplaces, and social groups, and the use of verbal aggression (e.g., foul language) and physical aggression to deal with interpersonal conflicts was commonplace [21]. On social media platforms, there was regular quarreling, doxing, and cyberbullying due to disagreement over political issues. More than one third (37%) of our participants had experienced victimization related to political disputes at least once in the past year. In addition, our findings indicated protesters, compared to non-protesters, experienced significantly more victimization by strangers. Given that protesters with different levels of reactive aggression had experienced victimization in one form or another, a positive relationship between victimization experiences and reactive aggression could not be observed.

Unexpectedly, victimization experiences related to political disputes by peers positively predicted the level of proactive aggression among non-protesters. It is unclear why aggression with a perceived political cause would increase the tendency of the victims who did not engage in the protests to use goal-driven aggression. Another interpretation of this finding is that non-protesters who used proactive aggression against their peer protesters were more likely to be aggressed by them, i.e., there were conflicts or fights between peers with contrary political views.

### 4.4. Narcissism

Narcissism significantly predicted a higher level of both proactive aggression and reactive aggression among protesters in Hong Kong. This is consistent with the notion that proactive aggression is motivated by the positive anticipation of one’s ability to perform aggressive acts and thereby obtain power and social status [16]. Proactive aggressors are frequently described as lacking empathy and remorse toward others due to their deliberate use of aggressive acts to dominate or intimidate [15]. With their focus on personal benefits, such as money and popularity, and insensitivity to punishment, they are likely to take advantage of others and engage in antisocial behaviors to demonstrate their power and sustain positive self-representations [44].

Research has suggested that narcissism is motivated by a desire to maintain one’s unrealistic, grandiose self-image by rejecting the negative perceptions of others [64,65]. Fossati et al. [66] found that overt narcissism was positively associated with both subtypes of aggression, whereas covert narcissism positively predicted reactive aggression only. Bolstered by inflated views of the self, protesters with narcissistic traits were more likely to be hypervigilant to ambiguous ego threats and to perceive themselves as victims, thereby justifying aggression towards others [64].

### 4.5. Impulsivity

Impulsivity significantly predicted a higher level of reactive aggression, but not proactive aggression, among the protesters. This is consistent with previous findings that reactive aggression is characterized by impulsive retaliation and emotional reactions to perceived threats, whereas proactive aggression is often planned with specific intentions and motivated by external reinforcements [12]. Our findings indicate that protesters with a high level of impulsivity were more likely to engage in risky and aggressive acts as immediate defensive or retaliatory responses to threats while overlooking the negative consequences of their behaviors.

### 4.6. Implications

Regarding the impacts of the results, the effect sizes ranged from 0.08 to 0.27 for the predictors in the final models. According to the interpretation guidelines by Funder and Ozer [67], the current findings are potentially consequential. There are implications at different levels.

First, those formerly developed, evidence-based interventions of reactive and proactive aggression could be applicable to the protesters [68,69,70]. Because of the political origin and the unprecedented scale of the social turmoil in Hong Kong, local healthcare professionals, and educators once had concerns over the effectiveness of existing intervention approaches. Nonetheless, our findings indicated protesters and non-protesters had similar psychosocial correlates of reactive and proactive aggression. The cognitive-behavioral approach, incorporated with the social information-processing (SIP) model [14], found to effectively reduce reactive and proactive aggression in adolescents [32], could be one of the intervention options.

Second, the development and implementation of systematic civic and moral education in the curriculum are recommended. Our results suggest that anger and moral disengagement are two major factors associated with reactive and proactive aggression among protesters. Shek [21] pointed out that vital topics, such as mutual respect and appropriate conflict resolution, are not adequately covered in the current curriculum in Hong Kong. Therefore, civic and moral education should be enhanced in school settings across different grades. Moreover, anger management skills should be strengthened through student counseling and other school programs. If young people had a better acquisition of moral values and moral reasoning, they would not use violence as a means to attain their goals or engage in the rationalization of their aggressive behaviors. If young people were better trained to manage their anger, they would be less impulsive and avoid turning to violence to release their negative emotions.

Third, at the family level, parents have a crucial role in reducing aggressive behaviors in their children during their development. Previous studies have revealed that parenting styles contributed to reactive and proactive aggression in Hong Kong children and adolescents [71]. Parenting styles and parental stress have also been found to be related to children’s psychopathic traits (e.g., impulsivity and narcissism) in the literature [72,73,74]. Parents should, therefore, be aware of their parenting style and, if necessary, adopt a healthier style. Moreover, they should not only focus on children’s academic performance but also on nurturing their moral and emotional development. Following Kohlberg’s cognitive-developmental theory of moral judgment [75], apart from school education, family education plays an important role in developing moral reasoning, especially at an early age. Hence, parents can prevent children and adolescents from developing moral disengagement and thus reduce their potential for subsequent aggressive or illegal behaviors.

Fourth, our study revealed that victimization was prevalent in the local community during 2019–2020, and that the perpetrators could be peers and strangers. Bullying among students is a reliable predictor of antisocial outcomes, such as delinquency, drug use, and psychopathy [76]. Victimization during adolescence has also been associated with an increased risk of developing internalizing and psychiatric problems and reduced social and occupational functioning in adulthood [77]. In the long term, aggression and victimization lead to adverse individual and societal outcomes, such as criminal offenses, substance abuse, unemployment, and negative impacts on physical and psychological well-being. These outcomes place a substantial financial burden on society. Despite these detrimental consequences, policymakers paid little attention to the issue of school violence even when Hong Kong ranked first among 75 regions around the world for the severity of school bullying [78]. In this context, the current findings can alert policymakers to the vast increase in aggression and victimization incidents among students after the Anti-Extradition Bill Movement.

Finally, given that a high level of anger towards the government and impulsivity could significantly predict reactive aggression, it is suggested that the government should take steps to develop diverse channels to understand the sources or reasons for the protesters’ anger and the perceived threats that the protesters faced, which triggered their violent acts in the Movement. By addressing their concerns and threats through enacting new policies or administrative measures, it could hopefully let citizens feel that the government is willing to repair its relationship with them through substantive actions, thereby reducing their level of anger and dissatisfaction towards the government and minimizing the possibility of protesters expressing their discontent through violent protests in the future.

### 4.7. Limitations

One major limitation of the current study is that the data were not collected from a random sample. The politically sensitive nature of the survey topic and the suspension of classes due to the COVID-19 pandemic resulted in few secondary schools and their students being willing to participate in the study. Although tertiary students were recruited openly on social media platforms with a financial incentive, it is unclear whether their interest in participation was a consequence of any of the psychological measures under investigation in the study. Demographic analyses also revealed few participants from Hong Kong Island, and none of the participating schools was located on Hong Kong Island. Moreover, although more than half of the participants did not report their family income, those that did declare income were predominantly middle class. About half of the respondents had a family income of >HKD 30,000, with the median monthly household income in 2019 in Hong Kong reported having been HKD 28,700 [79]. Furthermore, about 14% of the participants did not disclose whether they had participated in the protests. Therefore, our findings may not be generalizable to all adolescents and young adults who did or did not participate in the Anti-Extradition Bill Movement in Hong Kong. From our findings, the non-responses did not show any particular patterns of psychosocial measures similar to or distinctive from protesters or non-protesters. The non-responses were thus a mixed group of protesters and non-protesters.

Another limitation is that the current study is cross-sectional, and the findings cannot indicate causality. It remains possible that the anger and dissatisfaction, moral disengagement, and psychopathic traits resulted either from aggressive behaviors or from the negative social consequences of aggression (e.g., arrest, sentence, rejection by peers or family). As this study was designed and conducted after the major events of the Anti-Extradition Bill Movement, within-subject measures from the period preceding the Anti-Extradition Bill Movement could not be obtained.

In addition, some measures in the study were formulated regarding the local context, including the anger towards the government, satisfaction with the government, victimization by peers, and victimization by strangers. Although they were face valid, they did not undergo rigorous psychometric validation. There was a chance that some respondents comprehended the questions not as they were intended.

## 5. Conclusions

In conclusion, the present study revealed that young protesters in the 2019–2020 Hong Kong protests had a lower quality of life than those adolescents and young adults who had not participated in the Movement. The aggressive behaviors observed in the protests were mainly reactive. The protesters had similar psychosocial correlates of reactive and proactive aggression to the non-protesters despite the political causes. Future research and intervention efforts should focus on these psychosocial factors to tackle reactive and proactive aggression among adolescents and young adults in Hong Kong after the Movement to restore their quality of life.

## Figures and Tables

**Table 1:** Demographic Characteristics of Participants by Protest Participation.

Demographics		Protest Participation		
		Non-Protesters	Protesters	Non-Responses
Age	*M*	15.16	17.86	16.73
	*SD*	2.56	3.25	3.3
Sex	Female	99	291	59
	Male	188	308	82
Born in HK	No	48	88	15
	Yes	239	511	126
Area of Residence	Hong Kong Island	12	34	7
	Kowloon	176	257	72
	New Territories	94	304	60
	Missing	5	4	2
Family Income	<HKD 10,000	3	8	2
	HKD 10,000–19,999	25	55	8
	HKD 20,000–29,999	8	77	11
	HKD 30,000–39,999	11	53	5
	HKD 40,000–49,999	7	33	8
	≥HKD 50,000	29	67	7
	Receiving CSSA	9	7	1
	Unwilling/ Unable to Tell	179	185	76
	Missing	16	114	23

Note: CSSA = Comprehensive Social Security Assistance.

**Table 2:** Victimization Experiences Related to Political Disputes.

Perpetrator	Form	*n* ^a^	% ^a^	*M*	*SD*
Peers	Physical Victimization	78	8%	0.33	1.31
	Verbal Victimization	218	21%	0.74	1.80
	Social Manipulation	122	12%	0.45	1.50
	Attack on Property	58	6%	0.27	1.21
	Electronic Victimization	112	11%	0.45	1.54
	Overall Victimization	250	24%	0.45	1.28
Strangers	Physical Victimization	101	10%	0.42	1.47
	Verbal Victimization	247	24%	1.04	2.22
	Attack on Property	76	7%	0.33	1.33
	Electronic Victimization	153	15%	0.66	1.90
	Overall Victimization	275	27%	0.61	1.50

^a^ Number and percentage of participants who reported that they had experienced a form of victimization (rated ≥ 1 for the item) in the past year.

**Table 3:** Correlation of Two Subtypes of Aggression with Other Psychosocial Measures.

Measure	Reactive Aggression	Proactive Aggression
Reactive Aggression	-	0.44
Proactive Aggression	0.44	-
Anger towards the Government	0.26	0.12
Satisfaction with the Government	−0.23	−0.12
Victimization by Peers	0.13	0.12
Victimization by Strangers	0.22	0.18
Life Satisfaction	−0.13	−0.15
Moral Justification	0.34	0.27
Euphemistic Language	0.27	0.32
Advantageous Comparison	0.22	0.28
Diffusion of Responsibility	0.27	0.21
Displacement of Responsibility	0.23	0.23
Distorting Consequences	0.27	0.23
Attribution of Blame	0.26	0.20
Dehumanization	0.20	0.23
Impulsivity	0.47	0.32
Narcissism	0.47	0.35

Note: *n* = 1027. All correlation coefficients (*r_s_*) were significant at *p* < 0.001.

**Table 4:** Results of 2 × 3 ANCOVAs.

Measure	*F*			
	Age ^a^	Sex ^a^	Protest ^b^	Sex × Protest ^b^
Reactive Aggression	0.27	1.50	12.21 ***	0.94
Proactive Aggression	0.34	14.49 ***	2.30	0.44
Anger towards the Government	46.32 ***	3.71	248.72 ***	0.30
Satisfaction with the Government	30.33 ***	0.32	163.55 ***	0.81
Victimization by Peers	5.33 *	4.32 *	4.06 *	0.28
Victimization by Strangers	1.07	6.04 *	8.93 ***	2.54
Life Satisfaction	0.05	18.32 ***	6.36 **	3.44 *
Moral Justification	13.31 ***	23.81 ***	10.59 ***	0.45
Euphemistic Language	0.17	45.73 ***	6.35 **	1.34
Advantageous Comparison	5.34 *	19.41 ***	9.06 ***	0.99
Diffusion of Responsibility	4.83 *	1.34	8.67 ***	0.91
Displacement of Responsibility	1.66	15.06 ***	9.02 ***	0.26
Distorting Consequences	1.16	13.76 ***	5.46 **	1.52
Attribution of Blame	3.34	11.72 ***	3.49 *	2.61
Dehumanization	0.26	23.64 ***	9.97 ***	1.78
Impulsivity	5.02 *	4.88 *	8.79 ***	0.53
Narcissism	32.38 ***	11.57 ***	16.40 ***	2.75

Note: Protest = protest participation. Age is a covariate. ^a^ *df* = 11,020. ^b^ *df* = 21,020. * *p* < 0.05. ** *p* < 0.01. *** *p* < 0.001.

**Table 5:** Post hoc Comparisons among Protest Participation.

Measure	Non-Responses		Non-Protesters		Protesters		Differences ^a^	
	*EMM*	*SE*	*EMM*	*SE*	*EMM*	*SE*	Δ*EMM*	*d*
Reactive Aggression	3.51 ^b^	0.29	3.04 ^b^	0.22	4.32 ^c^	0.14	1.28	0.36
Proactive Aggression	0.74	0.16	0.42	0.12	0.73	0.08	0.31	-
Anger towards the Government	6.31 ^b^	0.23	3.58 ^c^	0.17	8.25 ^d^	0.11	4.67	1.68
Satisfaction with the Government	−2.95 ^b^	0.17	−1.28 ^c^	0.13	−4.07 ^d^	0.08	−2.79	−1.36
Victimization by Peers	0.59 ^b^	0.11	0.25 ^c^	0.08	0.48 ^bc^	0.05	0.24	-
Victimization by Strangers	0.72 ^b^	0.13	0.26 ^c^	0.10	0.74 ^b^	0.06	0.48	0.31
Life Satisfaction	162.54 ^b^	1.97	169.78 ^c^	1.50	163.81 ^b^	0.97	−5.97	−0.25
Moral Justification	2.08 ^bc^	0.16	1.75 ^b^	0.12	2.41 ^c^	0.08	0.66	0.34
Euphemistic Language	1.16 ^b^	0.12	0.73 ^c^	0.09	1.10 ^b^	0.06	0.37	0.25
Advantageous Comparison	0.92 ^b^	0.12	0.55 ^c^	0.09	1.01 ^b^	0.06	0.46	0.32
Diffusion of Responsibility	1.47 ^b^	0.14	0.95 ^c^	0.10	1.45 ^b^	0.07	0.50	0.30
Displacement of Responsibility	1.40 ^b^	0.13	0.88 ^c^	0.10	1.38 ^b^	0.06	0.50	0.31
Distorting Consequences	1.38 ^bc^	0.12	1.10 ^b^	0.09	1.48 ^c^	0.06	0.38	0.25
Attribution of Blame	1.46 ^bc^	0.12	1.18 ^b^	0.09	1.48 ^c^	0.06	0.30	0.20
Dehumanization	1.26 ^b^	0.12	0.61 ^c^	0.09	0.93 ^d^	0.06	0.32	0.22
Impulsivity	1.68 ^b^	0.15	1.64 ^b^	0.11	2.16 ^c^	0.07	0.52	0.28
Narcissism	1.89 ^b^	0.20	2.11 ^b^	0.15	2.92 ^c^	0.10	0.81	0.33

Note: *EMM* = estimate marginal mean. *EMM* with differing superscript were significant at the 0.05 level in Bonferroni post hoc tests. ^a^ Group differences between protesters and non-protesters. Cohen’s d was not calculated when there was no significant difference between protesters and non-protesters.

**Table 6:** Multiple Group Model Comparison.

Model	Reactive Aggression			Proactive Aggression		
	χ^2^	*df*	Unconstrained Path	χ^2^	*df*	Unconstrained Path
Null Model	50.34	18	Nil	138.62	18	Nil
Unconstrained Model	0.00	0	All	0.00	0	All
Alternative Model 1	26.29	17	SG	97.62	17	AC
Alternative Model 2	22.12	16	SG, Impulsivity	79.39	16	AC, VP
Alternative Model 3	-	-	-	63.61	15	AC, VP, Sex
Alternative Model 4	-	-	-	59.08	14	AC, VP, Sex, Narcissism

Note: AC = Advantageous Comparison. SG = Satisfaction with the Government. VP = Victimization by Peers. Both unconstrained models were just-identified models.

**Table 7:** Unconstrainted Model Predicting Reactive Aggression.

Factor	Protesters			Non-Protesters		
	β	*SE*	VIF	β	*SE*	VIF
Age	−0.10 **	0.03	1.16	−0.12 *	0.05	1.11
Sex	−0.11	0.06	1.19	−0.16	0.10	1.18
Reactive Aggression	-	-	-	-	-	1.60
Proactive Aggression	0.36 ***	0.03	1.25	0.35 ***	0.06	-
Anger towards the Government	0.08 *	0.04	1.38	−0.11	0.06	1.38
Satisfaction with the Government	−0.08 *	0.04	1.51	0.06	0.06	1.42
Victimization by Peers	0.01	0.05	2.10	0.02	0.08	3.02
Victimization by Strangers	0.02	0.05	1.99	−0.02	0.08	3.00
Life Satisfaction	−0.01	0.03	1.10	−0.06	0.05	1.15
Moral Justification	0.14 **	0.05	2.23	0.13	0.07	2.30
Euphemistic Language	0.08	0.06	2.75	−0.05	0.10	3.75
Advantageous Comparison	−0.10 *	0.04	2.07	−0.04	0.09	3.45
Diffusion of Responsibility	0.09 *	0.04	1.89	0.09	0.08	2.85
Displacement of Responsibility	−0.07	0.05	2.13	−0.08	0.08	2.87
Distorting Consequences	0.03	0.05	2.34	−0.03	0.08	3.01
Attribution of Blame	0.04	0.04	1.90	0.05	0.07	2.62
Dehumanization	−0.10 *	0.05	2.04	−0.05	0.07	2.86
Impulsivity	0.23 ***	0.04	1.78	0.27 ***	0.07	1.90
Narcissism	0.12 **	0.04	1.80	0.13	0.08	2.02

Note: VIF = Variance Inflation Factor. * *p* < 0.05. ** *p* < 0.01. *** *p* < 0.001.

**Table 8:** Final Multiple Group Model Predicting Reactive Aggression.

Factor	Protesters		Non-Protesters	
	β	*SE*	β	*SE*
Age	−0.12 ***	0.03	−0.09 ***	0.02
Sex	−0.13 *	0.05	−0.12 *	0.05
Proactive Aggression	0.35 ***	0.03	0.36 ***	0.03
Anger towards the Government	0.08 **	0.03	0.11 **	0.04
Satisfaction with the Government ^a^	−0.08 *	0.04	0.06	0.05
Victimization by Peers	0.01	0.04	0.01	0.04
Victimization by Strangers	0.02	0.04	0.01	0.03
Life Satisfaction	−0.02	0.03	−0.02	0.03
Moral Justification	0.15 ***	0.04	0.14 ***	0.04
Euphemistic Language	0.04	0.05	0.04	0.05
Advantageous Comparison	−0.10 *	0.04	−0.08 *	0.04
Diffusion of Responsibility	0.10 *	0.04	0.08 *	0.03
Displacement of Responsibility	−0.07	0.04	−0.07	0.04
Distorting Consequences	0.01	0.04	0.01	0.04
Attribution of Blame	0.04	0.04	0.04	0.04
Dehumanization	−0.09 *	0.04	−0.08 *	0.04
Impulsivity ^a^	0.24 ***	0.04	0.26 ***	0.05
Narcissism	0.13 **	0.04	0.11 **	0.03

^a^ The regression coefficients were significantly different between groups. * *p* < 0.05. ** *p* < 0.01. *** *p* < 0.001.

**Table 9:** Unconstrainted Model Predicting Proactive Aggression.

Factor	Protesters			Non-Protesters		
	β	*SE*	VIF	β	*SE*	VIF
Age	0.02	0.04	1.18	−0.04	0.04	1.13
Sex	0.31 ***	0.07	1.16	0.06	0.08	1.19
Reactive Aggression	0.42 ***	0.04	1.46	0.34 ***	0.07	1.55
Proactive Aggression	-	-	-	-	-	-
Anger towards the Government	−0.07	0.06	1.39	−0.02	0.05	1.40
Satisfaction with the Government	0.04	0.07	1.52	0.01	0.06	1.42
Victimization by Peers	0.09	0.08	2.08	0.08	0.09	3.01
Victimization by Strangers	−0.04	0.06	1.98	0.16	0.11	2.96
Life Satisfaction	−0.03	0.03	1.10	0.01	0.05	1.16
Moral Justification	−0.18 ***	0.04	2.22	−0.12	0.06	2.30
Euphemistic Language	0.07	0.05	2.76	0.15	0.09	3.72
Advantageous Comparison	0.13 *	0.06	2.07	0.32 *	0.12	3.27
Diffusion of Responsibility	0.01	0.05	1.90	0.00	0.07	2.87
Displacement of Responsibility	0.03	0.05	2.14	0.06	0.09	2.87
Distorting Consequences	−0.03	0.05	2.34	−0.13	0.08	2.98
Attribution of Blame	0.00	0.04	1.90	−0.02	0.07	2.62
Dehumanization	0.08	0.06	2.04	−0.03	0.07	2.86
Impulsivity	0.04	0.04	1.87	0.05	0.06	2.02
Narcissism	0.08 *	0.04	1.81	0.15 *	0.08	2.00

Note: VIF = Variance Inflation Factor. * *p* < 0.05. *** *p* < 0.001.

**Table 10:** Final Multiple Group Model Predicting Proactive Aggression.

Factor	Protesters		Non-Protesters	
	β	*SE*	β	*SE*
Age	0.00	0.03	0.00	0.02
Sex ^a^	0.30 ***	0.07	0.07	0.08
Reactive Aggression	0.39 ***	0.05	0.38 ***	0.05
Anger towards the Government	−0.04	0.04	−0.06	0.05
Satisfaction with the Government	0.02	0.04	0.03	0.05
Victimization by Peers ^a^	0.08	0.07	0.18 *	0.09
Victimization by Strangers	0.00	0.06	0.00	0.04
Life Satisfaction	−0.02	0.03	−0.02	0.03
Moral Justification	−0.18 ***	0.04	−0.16 ***	0.04
Euphemistic Language	0.10 *	0.05	0.10	0.05
Advantageous Comparison ^a^	0.14 *	0.06	0.27 ***	0.09
Diffusion of Responsibility	0.01	0.04	0.00	0.03
Displacement of Responsibility	0.05	0.05	0.04	0.04
Distorting Consequences	−0.06	0.05	−0.05	0.04
Attribution of Blame	−0.01	0.04	−0.01	0.04
Dehumanization	0.05	0.05	0.05	0.05
Impulsivity	0.04	0.04	0.04	0.03
Narcissism ^a^	0.09 *	0.04	0.13	0.08

^a^ The regression coefficients were significantly different between groups. * *p* < 0.05. *** *p* < 0.001.

## Data Availability

The author confirms that the data supporting the findings of the study are available within this article.
